# The use of hydrogel structures in production of extruded rice and investigation of its qualitative characteristics

**DOI:** 10.1002/fsn3.3466

**Published:** 2023-06-07

**Authors:** Sara Naji‐Tabasi, Mostafa Shahidi‐Noghabi, Atena Modiri Dovom, Maryam Davtalab

**Affiliations:** ^1^ Department of Food Nanotechnology Research Institute of Food Science and Technology (RIFST) Mashhad Iran; ^2^ Department of Food Chemistry Research Institute of Food Science and Technology (RIFST) Mashhad Iran

**Keywords:** composite hydrogel, extruded rice, gellan

## Abstract

The aim of this study was to investigate the quality parameters of extruded rice containing hydrogel and comparing with natural rice (Hashemi variety rice). Extruded rice was produced with composite hydrogel (gellan, xanthan and sodium alginate) at the concentrations of 0.0 (control sample), gellan (0.5%)‐alginate (0.5%) (GA1), gellan (1%)‐alginate (1%) (GA2), gellan (0.5%)‐alginate (0.5%)‐xanthan (0.1%) (GAX 1%), and gellan (1%)‐alginate (1%)‐xanthan (0.2%) (GAX2%). The use of hydrogels had no significant effect on moisture content, ash content, cooking time, and color properties of extruded rice (*p* ≥ .05). In contrast, hydrogel significantly increased water absorption ratio (WAR), water solubility index (WSI), water absorption index (WAI), and textural properties (*p* ˂ .05) of extruded rice. This observation supports the highest score found for extruded rice containing GA2% and GAX2% in sensory properties, which were similar to natural rice. GA2% rice sample showed the similar texture characteristic, cooking feature, and color parameter to natural rice, ultimately, showing better organoleptic properties.

## Graphical abstract

The hydrogel (GA1: gellan (0.5%)‐alginate (0.5%), GA2: gellan (1%)‐alginate (1%), GAX 1%: gellan (0.5%)‐alginate (0.5%)‐xanthan (0.1%), and GAX2%: gellan (1%)‐alginate (1%)‐xanthan (0.2%)) significantly increased water absorption ratio, water solubility index, water absorption index, and textural properties of extruded rice. GA2% rice sample showed similar texture characteristic, cooking feature, and color parameter to natural rice (Natural Hashemi Rice) and, ultimately, showed better organoleptic properties.

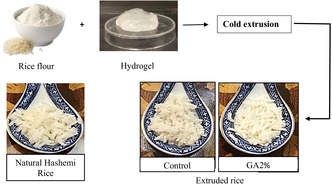

## 1 INTRODUCTION

Rice is one of the main sources of food in the most populated regions of the world, such as China, India, Indonesia, the Philippines, Japan, etc. It is a rich source of macro and micro nutrients because during the rice grinding process, fat and bran layers which include vitamin and micronutrients are removed and white rice is produced (Budi et al., 2016; Ding et al., 2022; Rhowell et al., 2021).

Recently, extruded rice is popular because it can be used as a carrier of nutrients. In addition, replacement of cereals flour by legumes can increase protein content of grain. The extruded rice has desirable properties such as more digestibility, smooth texture, whiteness, delicate taste, and low fracture rate. Also, it is noteworthy that the use of natural broken rice in the extrusion process reduces the price of the product (Dilrukshi et al., 2022; Li et al., 2021; Tas & Shah, 2021). Extruded rice products are of great interest to develop the production of more nutritional foods with lower glycemic index (Guan et al., 2023). Ingredients such as lubricants, colorants, flavorings, and binders play an important role to produce extruded products of desired physicochemical and rheological properties (Forsido et al., 2021; Nateghi et al., 2021).

The types of extruders are divided into hot and cold extruder. The cold extruder due to the lower temperature (below 100°C) of the fed chamber can decrease Millard reaction, disrupt the degradation of amino acids and vitamins, and reduce discoloration. Due to constant temperature in the cold extruder chamber, it is appropriate for shaping and mixing of food like meat products and pasta. The hot extruder, due to high temperature, can eliminate microorganism contamination so that the cold extruded product does not need packaging by special properties (Choton et al., 2020; Shelar & Gaikwad, 2019).

Hydrogels fabricated based on natural polymers have received attention for their excellent biocompatible properties, functionalization, easy gelation, and nontoxicity (Zhang et al., 2021, 2022). Hydrogels are applied in food as drug delivery, packaging, and recently for the manufacturing of structured foods (Naji‐Tabasi et al., 2023). Natural hydrocolloids are more interesting for creation hydrogels because of their various applications in domains such as delivery and creating structured foods. Researchers introduced hydrocolloids as one of the ingredients that have an effect on quality parameters like texture, rheological, cooking loss, and organoleptic properties of extruded rice. Hydrocolloids can create a three‐dimensional network that traps water molecules in this structure and prevent solid loss (Kraithong & Rawdkuen, 2020). Martínez et al. (2015) reported agar and carrageenan modified extruded wheat flour paste and gel properties. Zeng et al. (2021) found that xanthan and octenyl succinate anhydrate (OSA) starch improved by the twin‐screw extrusion co‐extruded XG‐OSA due to better rheological properties and that resistance to environmental stresses is suitable for dysphasia products (Zeng et al., 2021).

In this investigation, the characteristics of extruded rice formulation were improved with cooperation of composite hydrogel. The effects of type and concentration of composite hydrogel (gellan, alginate and xanthan) were studied on physical, textural, and organoleptic properties of extruded rice. The properties of the extruded rice sample were compared to natural rice as a popular variety (Hashemi variety rice).

## 2 MATERIALS AND METHODS

### 2.1 Raw material

Hashemi rice and rice flour were obtained from a local market (Hashemi variety, Rudsar, Iran). Xanthan gum and sodium alginate were purchased from Fufeng Company (Fufeng Company). Low acyl gellan was purchased from Qiaoli Co. (Qiaoli Co.). Sodium azide was obtained from AppliChem GmbH Co. (AppliChem GmbH Co.). Calcium chloride was obtained from Merck Co. (Merck Co.). Sugar, sodium chloride salt, monoglyceride, and corn starch were bought from a local market (Mashhad, Iran).

### 2.2 Hydrogel preparation

Hydrocolloid hydrogel was prepared using 0.5% w/w gellan‐0.5% %w/w alginate (GA1), 1% w/w gellan‐1% %w/w alginate (GA2) and 0.5% w/w gellan‐0.5% w/w alginate‐0.1% w/w xanthan (GAX 1%), and 1% gellan‐1% alginate‐0.2% xanthan (GAX 2%). The initial solution of gums was prepared by dissolving the desired hydrocolloid powder in deionized water at 80°C. Then, it was kept for 30 min in a hot water bath (80°C) for complete hydration. The gel structure was formed by adding 0.1 M calcium chloride solution (0.25% w/v) (Naji‐Tabasi et al., 2023).

### 2.3 Extrusion process

To produce extruded rice, broken rice flour was used via the cold extruder method under a certain pressure condition at 40°C. Rice dough ingredients (based on flour weight) were as follows: 2% calcium chloride, 0.5% sodium chloride, 0.65% emulsifier, 0.5% sugar, and 15% corn starch mixed in dough mixer. Then, 60% hydrogel (based on flour weight) was added to the mixture. The dough was treated under a steam injection condition for 10 s (2 times). The total dough was kneaded for 10 min. Furthermore, it was put under pressure for feeding into barrel of extruder. Extruded product was dried at 40°C under air flow for ~6 h. After drying, extruded rice was immediately cooled to ambient temperature (25°C) and packed. In order to determine the hydrogel effect on rice grain properties (including GA1, GA2, GAX1, and GAX2), a control sample without hydrogel was prepared.

The quality properties of natural Hashemi rice were compared with the extruded rice.

### 2.4 Moisture content

The moisture content was determined in triplicate according to the method described in AOAC (AOAC, 2005). After weighing, the samples were placed in an oven at 105°C to reach a constant weight and then placed in the desiccator. The moisture content was calculated as the difference between initial weight and final weight (AOAC, 2005).

### 2.5 Ash content

The extruded rice sample (3 g) was poured into silica crucibles and incinerated on the burner. Then charred samples were heated in the furnace (Excition) at 550°C until the sample turned completely white. The samples were weighed after cooling in a desiccator. The ash concentration percentage was calculated as the difference between initial weight and final weight (AOAC, 2005).

### 2.6 Cooking quality

#### 2.6.1 Optimum preparation time

Optimum preparation time (OPT) was determined in triplicate. Five grams of rice grains were hydrated in 150 mL boiling water. The OPT was matched to the moment of disappearance of the starchy core and soft tissue of the grains (Patria et al., 2020).

#### 2.6.2 Water absorption ratio

The water absorption ratio (WAR) was determined according to Patria et al. (2020) in triplicate. The hydrated samples after OPT were immediately cooled (20°C) and weighed. The water absorption capacity was calculated as follows:

(1) $$\mathrm{WAR}\;\left(\%\right)=\frac{\left(W_{2}-W_{1}\right)\times 100\;}{W_{1}\;}$$

*W*_1_ was weight of dry grain rice before cooking (OPT) and *W*_2_ was weight of grains after cooking (Patria et al., 2020).

#### 2.6.3 The water absorption index (WAI) and the water solubility index (WSI)

Water absorption index and WSI were determined according to the method described by Patria et al. (2020) in triplicate. The sample flour (2 g) was diluted in 20 mL of distilled water and poured in centrifuge tubes. The solution was centrifuged at 865 *g* for 20 min. Then, the supernatant was poured in an aluminum container and dried in oven at 105°C for 24 h. The weight of the gel and dried supernatant was recorded. WAI and WSI were calculated as follows (Equations 2 and 3; Patria et al., 2020):

(2) $$\mathrm{WAI}\;\left(g/g\right)=\left[\frac{\text{Weight of}\;\mathrm{gel}\;\left(g\right)\;}{\text{Weight of flour}\;\left(g\right)}\right]$$

(3) $$\mathrm{WSI}\;\left(g/g\right)=\left[\frac{\text{Weight of}\;\mathrm{dry}\;\text{matter in supernatant}\;\left(g\right)\;}{\text{Weight of flour}\;\left(g\right)}\times 100\right]$$

#### 2.6.4 Cooking loss

After cooking in OPT, 5 g of rice grain was rinsed with 100 mL of distilled cold water (20°C). Both the hydration water from cooking and the rinsing water were collected in a container and then evaporated in an oven at 105°C to constant weight. Cooking loss was calculated as the difference between weight of dry matter in cooking water (g) and initial weight of rice grain (g) (Patria et al., 2020).

#### 2.6.5 Lateral expansion

Lateral expansion was measured using the length of rice before and after cooking in OPT. Rice sample diameters were measured by a digital caliper once they were cooled (Saadat et al., 2019).

#### 2.6.6 Textural characteristics of rice

The rice grain before and after cooking was evaluated by a texture analyzer (TA‐XT Plus Texture Analyzer). The cutting test was performed with blade probe (BLADE, HDP/BSW) at a test speed of 1 mm/s to retain 50% sample form. The measurement of hardness and adhesiveness of cooked and uncooked rice was performed at ambient temperature (25°C) (Bouasla & Wójtowicz, 2021).

#### 2.6.7 Color properties

The images of dry and cooked rice were scanned by a scanner (Canon scanner, LiDE220). Then Image J software (version 1.48 a) was used to measure color factors. The color properties of the samples were examined in the Lab color space. The values are L* (the luminance (0) or lightness (0–100)), a* (redness (+120) to greenness (−120)) and b* (yellowness (+120) to blueness (−120)) (Sun, 2016). ∆*E* was calculated according to Equation 4 as a measure of the color difference between the extruded product and the Hashemi rice (Xiao et al., 2022).

(4) $$∆E=\sqrt{{\left(L_{2}-L_{1}\right)}^{2}+{\left(a_{2}-a_{1}\right)}^{2}+{\left(b_{2}-b_{1}\right)}^{2}}$$

#### 2.6.8 Sensory analysis

Sensory evaluation of rice was conducted after cooking in OPT. Sensory analysis including color, aroma, texture, chewability, and overall acceptability was done with 22 trained panelists (the age range of 24–40 years). The panelists drank warm water during the test interval for each sample. The 5‐point hedonic scale was used for sensory analysis (Naji‐Tabasi et al., 2021).

### 2.7 Statistical analysis

The results were analyzed by a completely randomized factorial design according to ANOVA. Then Duncan's multiple range test was applied to obtain the significant difference between means (*p* ≤ .05).

## 3 RESULT AND DISCUSSION

### 3.1 Moisture content

Results showed no statistical significance (*p* > .05) between the moisture content of the hydrogel restructured rice compared to the control (restructured rice sample without hydrogel) (data not shown). According to experiment data, GA2% and control rice had the lowest moisture content, while the highest amount was observed in GA1% and GAX1% (*p* > .05). The moisture content of the rice samples increased with the increment of gellan gum concentration (*p* > .05). But alginate gum content had no influence on moisture content. Maybe, the negative charges on the alginate gum surface led to a delay in gelatinization and starch swelling. The anionic gums, such as xanthan, alginate, carrageenan, and Arabic gum, can delay gelatinization and prevent the disappearance of amylose from the dough (Ranjbar et al., 2018).

Ranjbar et al. (2018) studied the effect of guar and Arabic gum concentration (0.1%, 0.2%, 0.3% and 0.4% w/w) on the properties of extruded rice. Their results showed that physicochemical properties, such as moisture content, water solubility index, water absorption index, and apparent density, in samples containing different concentration of guar gum were more than in samples containing of Arabic gum. Also, initial moisture content increased by 30% compared to the samples without gum (Ranjbar et al., 2018). Saadat et al. (2019) reported guar gum and Arabic gum (0.5% and 0.1%) increased the moisture content of the extruded rice compared to the control samples (Saadat et al., 2019).

### 3.2 Ash content

The hydrogel addition had no significant effect on the ash content of extruded rice (1.80%–2.25%) in comparison to the control sample (1.85%) (*p* > .05) (data not shown). However, the ash content of samples including GAX was more than other samples. Saadat et al. (2019) reported guar and Arabic gum (0.5% and 0.1%) increased the ash content of the samples which is caused due to the mineral ingredients in these gums (Saadat et al., 2019). Patria et al. (2021) reported galactomannan increased ash content of restructured rice using extruder (Patria et al., 2021).

### 3.3 Cooking time

**FIGURE 1:**
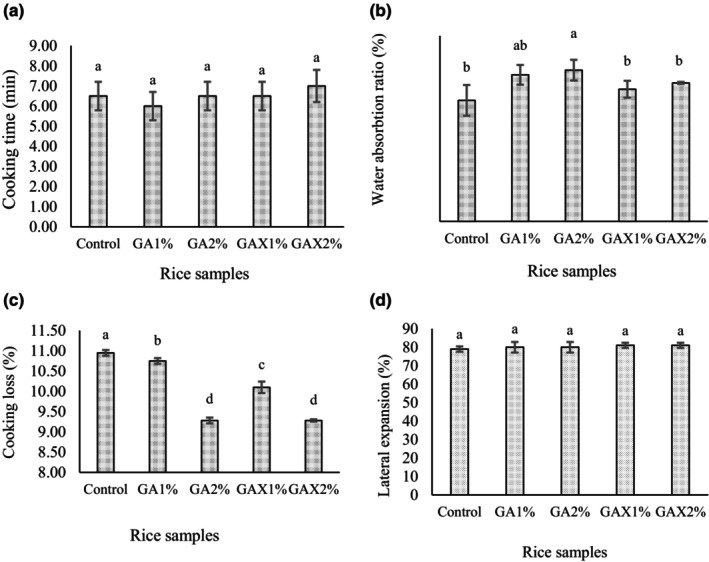
Cooking properties of rice samples containing different concentrations of hydrogel (GA, gellan‐alginate; GAX, gellan‐alginate‐xanthan) (cooking time (min) (a), water absorption ratio (%) (b), cooking loss (%) (c), and lateral expansion (d)).

Figure 1a showed that the extruded rice containing hydrogels had no significant difference with cooking time (6–7 min) when compared to control sample without hydrogels (6.50 min) (*p* ˃ .05). However, it was expected that the presence of hydrocolloids delay the gelatinization of starch granules. Hydrocolloids prevent starch gelatinization and swelling due to the covering of starch grains. Gelatinization of tapioca was delayed due to the repulsion between starch and gum grains (both negatively charged). These studies made it clear that factors such as the molecular structure of hydrocolloids and the charges of starch and hydrocolloids have an important role in product rheological properties. The damaged starch leads to more starch granulation, swelling, and more moisture absorption (Chaisawang & Suphantharika, 2006). Maybe, using hydrogel instead of gum powder in formulation has a different effect, as there is no competition between hydrogel and starch in absorbing water.

### 3.4 Water absorption ratio

The water absorption ratio in extruded rice including GA2 (326%) was significantly higher than rice without hydrogel (261%) (*p* ˂ .05), while other treatments had no significant difference compared to the control sample (*p* ˃ .05) (Figure 1b). This could possibly explain that the gellan and alginate gums can increase water absorption in the extruded sample more than xanthan gum. However, extrusion processing has an important effect on gelatinization of rice starch granule so that when the rice is heated, grains rapidly absorb moisture (Detzel et al., 2016). Also reported is that due to the gums' intrinsic water absorption, using them in dough can affect the water absorption ratio, swelling, and gelatinization of grains (Srikaeo et al., 2018).

### 3.5 Cooking loss

The effect of the gellan, alginate, and xanthan gum mixture in different concentrations on the cooking loss of extruded rice is presented in Figure 1c. The result showed that all of the treatments (9.28%–10.75%) had a significant effect on the reduction of the loss cooking compared to the restructure sample without hydrocolloid (10.95%) (*p* ≤ .05). Nevertheless, GA2% and GAX2% treatments (9.28%) significantly decreased solid loss (*p* ≤ .05). The higher cooking loss of samples including lower concentration (GA1% and GAX1%) or without hydrocolloids (control sample), indicates more leaching out during extruded rice cooking, which can increase the turbidity of cooking water. More hydrogen bonding between hydrocolloids and starch molecules can decrease cooking loss (Ismail et al., 2017; Kraithong & Rawdkuen, 2020; Wang et al., 2016). Kraithong and Rawdkuen (2020) investigated the effect of guar, carboxymethyl cellulose, and xanthan gum on extruded rice. The results showed lower cooking loss in sample including guar gum and carboxymethyl cellulose compared to that of xanthan. They explained lower cooking loss may be due to less expansion ratio and higher crystallinity of hydrocolloids (Kraithong & Rawdkuen, 2020).

### 3.6 Lateral expansion

The lateral expansion results are shown in Figure 1d. The hydrocolloids had no significant effect on lateral expansion of restructured rice compared to that of the control sample (79%–81%) (*p* ˃ .05). Although, the results showed higher lateral expansion in extruded rice containing hydrocolloids compared to control sample. Gelatinization of hydrocolloids decreases the bulk density and elasticity and as a result increases the lateral expansion (Awolu & Akintade, 2021; Mirzaei et al., 2021; Naji‐Tabasi & Mohebbi, 2015). In this present study, it was observed that samples containing GA and GAX slightly increased the height of rice. Although fat protects starch grains against gelatinization to reduce their gelatinization, expansion decreased (Boopathy et al., 2021; Yoo et al., 2013).

In general, as the temperature of the extruder chamber increases, the bulk density decreases as a result of the elastic force and insufficient vapor pressure. Electron microscope images showed that by increasing the extrusion temperature, looser granules are formed inside the molten material, which is able to create a microporous structure with gas bubbles, which ultimately reduces the apparent density (Gulati et al., 2020; Zhuang et al., 2010).

### 3.7 Water absorption and solubility index (WSI)

**FIGURE 2:**
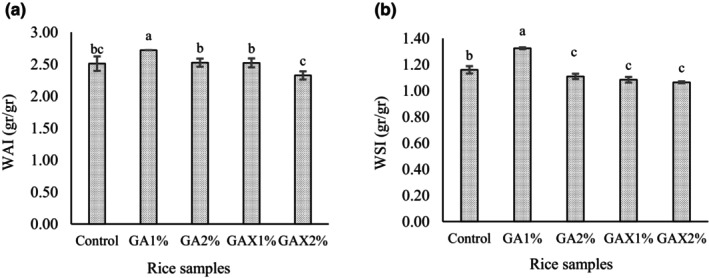
Water absorption index (g/g) (a) and water solubility index (g/g) (b) of rice samples containing different concentration of hydrogel (GA, gellan‐alginate; GAX, gellan‐alginate‐xanthan).

The highest value of water absorption index was observed in GA1 sample (2.72 g/g) and the lowest in GAX2 (2.33 g/g) and control (2.51 g/g) (Figure 2a). Loubes et al. (2016) reported guar gum increased water absorption (58.7%) of modified rice compared to xanthan gum (Loubes et al., 2016). Also, Mirzaei et al. (2021) reported that adding hydrocolloids such as carboxymethylcellulose and locust bean gums in flour increased water absorption of extruded rice (Mirzaei et al., 2021).

The highest water solubility index (WSI) value was obtained in GA1 treatment (1.33 g/g) (Figure 1b). The alginate and gellan gum increased WSI in the GA1% extruded sample compared to the control (*p* ≤ .05). The WSI value coresponding to starch molecule behaviour during gelainazation and play important role in leaching. Intrinsically, amylose plays a more important role in water solubility index than amylopectin (Cai et al., 2015). Sandhu et al. (2015) reported gums decreased leaching out the amylose and amylopectin during gelatinization. These authors explained that the solubility index decreased due to gums complex with amylose. Nevertheless, their result showed leaching out of starch compound in cooked products including guar gum was more compared to that of xanthan gum. These authors attributed this result due to repulsing between starch granules phosphate groups and negative charge of anionic gum. In addition, gums can absorb water in a swell granule that decreased both starch swelling and water absorption of the rice grain (Raungrusmee et al., 2020; Sandhu et al., 2015).

### 3.8 Color attributes

**FIGURE 3:**
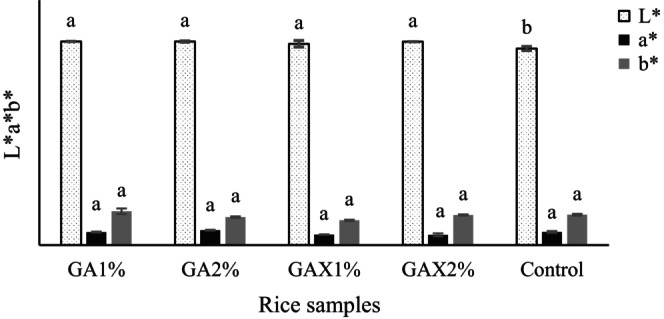
Color parameters of extruded rice samples containing different concentration of hydrogel (GA, gellan‐alginate; GAX, gellan‐alginate‐xanthan).

The color attribute of rice grain was analyzed according to L*, a*, and b* space. As shown in Figure 3, lightness of all the extruded samples including hydrogels was significantly (*p* ≤ .05) higher than the control sample. Naji‐Tabasi and Mohebbi (2015) reported using hydrocolloids increases water distribution that affects Maillard and caramelization reactions, thus increasing the lightness of products. The results showed no considerable difference in a* and b* index of all the extruded samples (*p* ≤ .05).

### 3.9 Textural properties

The textural properties results of samples before and after cooking are shown in Table 1. In terms of samples before cooking, the control rice hardness (1108.75 g) was significantly higher than other samples containing hydrogel (436.65–637.39 g) (*p* ≤ .05). Temperature can increase the degree of crystallinity of extruded rice and organization of amylose complex with other ingredients that, in this case, due to the cooking temperature, hydrocolloids had a strong intermolecular bonding with starch molecules which increased rice hardness after cooking (Budi et al., 2016; Yang et al., 2020). After cooking, hardness of samples containing GA1% (8.66 g) and GA2% (8.87 g) was higher than other extruded samples (*p* > .05). In the other word, xanthan in the hydrogel restructure rice decreased texture hardness. These results may be due to lower solid loss and higher crystallinity of the rice noodle in which starch chain is stronger (Jafari et al., 2018; Kraithong et al., 2019). Liu et al. (2022) reported when the inulin were added to extruded mixture, it bound to water molecules through hydrogen bonds. In this way, inulin act as stabilizer and decreased mobility of water molecules between other molecules and decreased water loss of extruded flour. It is hypothesized that the reduced water accessibility of starch would increase the cross‐links between starch chains and non‐starch materials, which would subsequently increase hardness (Liu et al., 2022). As reported in WAR results, GA2% and GA1% had higher water absorption compared to other treatments in this work. Li et al. (2017) found adhesiveness is due to leaching of starch molecules and decreasing crystalline structure. Also, they confirmed that heat can disrupt the network structure and increase the leaching of starch molecules (Li et al., 2017).

**TABLE 1:** Textural properties of extruded rice before and after cooking.

Rice samples	After cooking		Before cooking
	Hardness (g)	Adhesiveness (g.s)	Hardness (g)
Control sample	6.83 ± 0.44 ab	0.57 ± 0.21 a	1108.75 ± 17.04 a
GA 1%	8.66 ± 0.20 b	0.63 ± 0.52 a	585.53 ± 0.16 c
GA 2%	8.87 ± 0.41 b	0.31 ± 0.42 a	521.54 ± 15.91 d
GAX 1%	6.69 ± 0.68 c	0.60 ± 0.11 a	436.65 ± 17.15 e
GAX 2%	7.75 ± 0.99 bc	0.89 ± 0.32 a	637.39 ± 33.14 b
Hashemi rice	14.65 ± 1.30 a	0.36 ± 0.44 a	633.89 ± 22.45 a

*Note*: Means ± standard deviation. Different letters in each column indicate statistically significant differences (*p* ≤ .05).

### 3.10 Sensory properties

Sensory properties of extruded rice containing hydrogels (GA1%, GA2%, GAX1%, and GAX2%) are shown in Table 2. In terms of color characteristics, there was no significant difference between the samples (4.10–4.78) (*p* > .05). The control sample and GAX1% obtained the lowest scores in terms of appearance, taste, aroma, and texture, although the difference was not significant (*p* > .05). As a result, the lowest overall acceptance score was obtained by the control (3.20) and GA 1% (3.30) samples (*p* ≤ .05). In a similar study, Raungrusmee et al. (2020) reported xanthan in gluten‐free noodles had the highest score in overall acceptability. Also, it was reported that gellan, carrageenan, and glucomannan improve mouthfeel of rice starch (Huang et al., 2007). Ranjbar et al. (2018) found that guar and Arabic gums increase the overall acceptability of extruded rice.

**TABLE 2:** Sensory properties of extruded rice and Hashemi rice.

Sample	Color	Appearance	Aroma	Taste	Texture	Overall acceptability
Control	4.10 ± 0.74 a	3.50 ± 0.85 a	4.20 ± 0.63 a	3.90 ± 0.74 a	3.50 ± 0.48 a	3.20 ± 0.36 b
GA 1%	4.33 ± 0.67 a	3.60 ± 0.52 a	3.80 ± 0.42 a	3.70 ± 0.67 a	3.80 ± 0.42 a	3.30 ± 0.42 b
GA 2%	4.67 ± 0.50 a	4.40 ± 0.70 a	4.60 ± 0.52 a	4.40 ± 0.50 a	4.30 ± 0.63 a	4.40 ± 0.48 a
GAX 1%	4.78 ± 0.48 a	3.78 ± 0.83 a	4.00 ± 0.28 a	4.20 ± 0.63 a	4.10 ± 0.74 a	4.20 ± 0.48 a
GAX 2%	4.70 ± 0.42 a	4.40 ± 0.53 a	4.50 ± 0.53 a	4.30 ± 0.52 a	4.20 ± 0.67 a	4.30 ± 0.74 a
Hashemi rice	4.65 ± 0.13 a	0.61 a ± 4.38	0.65 a ± 4.75	0.78 a ± 4.69	0.88 a ± 4.88	0.55 a ± 4.60

*Note*: Means ± standard deviation. Different letters in each column indicate statistically significant differences (*p* ≤ .05).

### 3.11 Comparison of selected restructured rice with Hashemi rice

In order to develop extruded rice containing hydrogel as a substitute product for natural rice, selected samples (GA2% and GAX2%) were selected and compared to Hashemi rice.

In compared to Hashemi rice color characteristics, extruded rice samples containing GA2% and GAX2% had the higher lightness and lower a* and b* (Table 3). This confirms that non‐starch hydrocolloid distribute water in the starch structure and create lighting grain. Total color difference (∆*E*) values between GA2% and GAX2% with Hashemi rice were 12.13 and 12.20, respectively (Table 3). Producing sample through cold extruder causes low color difference in compared with the original rice.

**TABLE 3:** Cooking and color properties of optimum extruded rice and Hashemi rice.

Rice properties	Extruded rice GA2%	Extruded rice GAX2%	Hashemi rice
Cooking time (min)	6.50 ± 0.71 b	7.00 ± 0.00 b	15.00 ± 2.49 a
Water absorption ratio (%)	326 ± 22.23 a	299.00 ± 2.12 b	201.50 ± 8.59 c
Cooking loss (%)	9.28 ± 0.07 a	9.29 ± 0.03 a	9.21 ± 1.16 a
Lateral expansion	80.00 ± 2.83 a	81.10 ± 1.42 a	80.43 ± 2.23 a
L*	99.77 ± 0.26 a	99.77 ± 0.01 a	88.65 ± 0.74 b
a*	7.25 ± 0.26 ab	4.98 ± 0.76 b	8.45 ± 0.89 a
b*	13.73 ± 0.35 c	14.77 ± 0.24 b	18.42 ± 1.93 a
∆*E*	12.13 ± 0.40 a	12.20 ± 0.90 a	‐

*Note*: Means ± standard deviation. Different letters in each row indicate statistically significant differences (*p* ≤ .05).

In terms of cooking characteristics, the extruded samples had lower cooking time, which was due to the degradation of starch structure in extruder (Table 3). The water absorption ratio increased due to more water absorption of hydrogel in restructured rice, where the water absorption ratio of extruded rice containing GA2% was highest (326%) and the Hashemi rice was lowest (201%) (Table 3). In terms of cooking loss, there is no significant difference between the selected extruded rice samples (9.29% and 9.28%) and Hashemi rice (9.21%). About lateral expansion after cooking, the height of optimum extruded grains (81% and 80%) was similar to Hashemi rice (80%).

Hashemi rice had the highest hardness in the extruded rice (Table 1). The extruded rice containing GA2% had higher stiffness (8.87 g) value compared to Hashemi rice and GAX2%. However, there was no significant difference before cooking (*p* ˃ .05) in hardness of selected extruded rice samples (637.39 and 521.54 g) and Hashemi rice (633.89 g). The amount of adhesiveness in the GAX2% sample was more than the other samples (Table 1), which due to increment in gums, adhesiveness increased.

The result showed no significant difference between the sensory characteristics of optimum extruded samples and Hashemi rice (Table 2). Nevertheless, our result showed that extruded rice containing GA2% sample can be a good alternative for Hashemi rice.

## 4 CONCLUSION

According to the results, addition of different levels of gellan‐alginate and gellan‐alginate‐xanthan gum hydrogel network had different effects on the texture, color, and physical parameters of the produced structured rice. The use of hydrogels in the rice structure significantly reduced the cooking loss and increased the water absorption ratio. In terms of hardness after cooking, GA2% and GA1% treatments had higher values (*p* < .05). With the presence of xanthan gum in the hydrogel structure, the hardness of rice decreased. The structure of xanthan‐containing hydrogels had lower hardness than other hydrogels. Comparison of structured rice samples with Hashemi rice samples also showed that in terms of cooking characteristics, structured samples have less cooking time and stiffness. But they had more water absorption and desirable color. Investigation of sensory properties did not show a significant difference between structured samples and Hashemi rice. According to the results, structured rice containing GA2% and GAX2% hydrogels improved acceptable quality.

## AUTHOR CONTRIBUTIONS

**Sara Naji‐Tabasi:** Conceptualization (equal); data curation (equal); methodology (equal); project administration (equal); supervision (equal); validation (equal); writing – review and editing (equal). **Mostafa Shahidi‐Noghabi:** Data curation (equal); methodology (equal); project administration (equal); supervision (equal); writing – review and editing (equal). **Atena Modiri Dovom:** Investigation (equal); software (equal). **Maryam Davtalab:** Formal analysis (equal); software (equal); writing – original draft (equal).

## CONFLICT OF INTEREST STATEMENT

The authors declare that they do not have any conflict of interest.

## Data Availability

All data generated or analyzed during this study are available from the corresponding author on reasonable request.
